# *PTEN* loss is not a determinant of time to castration-resistance following androgen-deprivation therapy in prostate cancer: a study from Jordan

**DOI:** 10.25122/jml-2023-0034

**Published:** 2023-04

**Authors:** Abdallah Alzoubi, Samir Al Bashir, Aya Smairat, Ahmad Alrawashdeh, Husam Haddad, Khalid Kheirallah

**Affiliations:** 1 Department of Pathological Sciences, College of Medicine, Ajman University, Ajman, United Arab Emirates; 2 Department of Pharmacology, Faculty of Medicine, Jordan University of Science and Technology, Irbid, Jordan; 3 Department of Pathology and Microbiology, Faculty of Medicine, Jordan University of Science and Technology, Irbid, Jordan; 4 Department of Physiology and Biochemistry, Faculty of Medicine, Jordan University of Science and Technology, Irbid, Jordan; 5 Department of Allied Medical Sciences, Faculty of Applied Medical Sciences, Jordan University of Science and Technology, Irbid, Jordan; 6 Department of Pathology and Laboratory Medicine, Ministry of Health, Amman, Jordan; 7 Department of Public Health and Community Medicine, Faculty of Medicine, Jordan University of Science and Technology, Irbid, Jordan

**Keywords:** *PTEN*, castration resistance, CRPC, ADT, prostate cancer, Jordan

## Abstract

Androgen deprivation therapy (ADT) remains the principal treatment of advanced prostate cancer. However, most patients eventually experience treatment failure, resulting in castrate-resistant prostate cancer (CRPC). Loss of the tumor suppressor gene phosphatase and tensin homolog (*PTEN*) has been linked to poor survival in prostate cancer. We have recently shown that *PTEN* loss is evident in approximately 60% of prostate cancer cases in Jordan. However, the correlation between *PTEN* loss and response to ADT remains unclear. This study aimed to determine the relationship between *PTEN* loss and time to CRPC in Jordan. We conducted a retrospective analysis of confirmed CRPC cases at our institution from 2005 to 2019 (N=104). *PTEN* expression was assessed using immunohistochemistry. Time to CRPC was calculated from the initiation of ADT to the confirmed diagnosis of CRPC. Combination/sequential ADT was defined as the use of two or more classes of ADT concomitantly or switching from one class to another. We found that *PTEN* loss was evident in 60.6% of CRPC. Mean time to CRPC was not different between patients with *PTEN* loss (24.8 months) and those with intact *PTEN* (24.2 months; p=0.9). However, patients receiving combination/sequential ADT had a significantly delayed onset of CRPC compared to patients on monotherapy ADT (log-rank Mantel-Cox p=0.000). In conclusion, *PTEN* loss is not a major determinant of time to CRPC in Jordan. The use of combination/sequential ADT procures a significant therapeutic advantage over monotherapy regimens, delaying the onset of CRPC.

## INTRODUCTION

Prostate cancer is a principal cause of cancer-related morbidity and mortality among men worldwide [1]. In 2017, it was reported that prostate cancer, together with lung and colorectal cancers, represent almost 30% of the cancer burden in men in the Eastern Mediterranean region [2]. These rates were approximately 10% lower than those reported globally by the Global Burden of Disease study published in 2016 [3]. The exact cause of this ethnic disparity in prostate cancer prevalence rates remains unclear. To investigate whether differences in the expression levels of key oncogenes or tumor suppressor genes could explain this disparity, we recently examined the expression of the tumor suppressor gene phosphatase and tensin homolog (*PTEN*) in a cohort of prostate cancer patients from Jordan, a country of predominantly Arab population in the Eastern Mediterranean region. We found that the frequency of *PTEN* loss was higher than rates reported from several other cohorts of Eastern Asian countries and comparable to rates in Western countries [4]. The precise mechanism(s) underlying this ethnic variation in *PTEN* loss prevalence is yet to be determined.

*PTEN* is a tumor suppressor that tightly regulates the phosphoinositide-3-kinase/serine-threonine kinase/mammalian target of rapamycin (PI3K/AKT/mTOR) signaling axis, which is the main survival pathway involved in prostate cancer [5]. *PTEN* mutations were reported in over 50% of metastatic prostate cancers [6], accounting for poor disease prognosis, early biochemical recurrence, and androgen independence [7]. Despite the extensive evidence on the prognostic value of *PTEN* mutations in prostate cancer, their correlation with treatment outcomes remains a rich area of investigation.

Androgens play a pivotal role in maintaining normal prostate gland homeostasis and function by acting through the androgen receptor (AR), also known as the nuclear receptor subfamily 3 group C, gene 4 (NR3C4). Conversely, AR is critically involved in the molecular mechanisms of the initiation and progression of prostate cancer [8]. This involvement is most evident because androgen-deprivation therapy (ADT), which aims to reduce androgens or prevent them from promoting prostate cancer cell growth, remains the primary treatment for almost all locally advanced and metastatic prostate cancers [9]. To achieve ADT, several classes of drugs targeting the production of androgens are currently employed. These drugs either (a) reduce the production of androgens, such as the luteinizing hormone-releasing hormone (LHRH) agonists (e.g., goserelin and triptorelin), or antagonists, (b) inhibit the synthesis of adrenal and intra-tumoral androgens (e.g., abiraterone), or (c) block the activity of the AR (e.g., bicalutamide). Combining these drug classes is often indicated, particularly to counteract the transitional testosterone flare observed at the beginning of treatment with LHRH agonists [10].

While most patients with prostate cancer initially respond to ADT, nearly all eventually experience treatment failure, resulting in castrate-resistant prostate cancer (CRPC) [11]. This resistance is defined as disease progression during ADT by either rising serum prostate-specific antigen (PSA) levels despite low levels of circulating testosterone (<50 ng/dl), development of symptoms in the presence of pre-existing cancer, or detecting new malignant lesions [12]. The exact mechanism of ADT resistance is still not clear. However, several mechanisms have been postulated, including amplification of the AR locus and somatic mutations of the tumor, which were evident in almost 90% of metastatic CRPC [13]. Moreover, loss of *PTEN* function has been linked to increased AR activity, even at low levels of circulating testosterone, ultimately leading to ADT failure and poor prognosis [14].

Considering the high prevalence of *PTEN* mutations in prostate cancer cases in Jordan and the scarcity of data on the independent correlation between *PTEN* expression status and ADT response, this follow-up study aimed to determine the relationship between *PTEN* loss and time to castration-resistance following ADT in a prostate cancer cohort from Jordan.

## Material and Methods

This was a retrospective analysis of confirmed prostate cancer cases at King Abdullah University Hospital (KAUH). Hospital records of patients were accessed through the Department of Pathology at KAUH, and data were collected for all patients enrolled in the study from 2005 to 2019.

### Study population

Hospital records of Jordanian patients with a confirmed diagnosis of CRPC following ADT (n=104) were retrieved. CRPC was defined as rising PSA levels, on 2 one-week-apart readings, despite low circulating testosterone levels (<50 ng/ml) during the course of ADT. Baseline PSA levels, as well as the highest PSA level during treatment, were recorded. Time-to-resistance was estimated as the time between the starting date of ADT and the date of confirmed laboratory diagnosis of CRPC.

### Sample collection

Prostate cancer samples were initially collected by transurethral resection of the prostate (n= 29) or needle core biopsy (n= 75). All prostate cancer samples were reviewed by two pathologists (SA and HH), and prostate cancer was classified according to the guidelines of the 2005 and 2014 WHO-International Society of Urological Pathology (ISUP) Consensus conferences [15]. All prostate cancer cases used in this study were diagnosed as prostatic adenocarcinoma, acinar type. No ducal or mixed (acinar/ ductal) adenocarcinoma cases were included in our cohort.

### Immunohistochemistry evaluation of *PTEN*

Two to four representative sections from each prostate cancer specimen demonstrating the greatest volume of the tumor were selected for immunohistochemistry (IHC) evaluation of *PTEN*. IHC was performed on 4-µm prostate sections using a Dakoautostainer-Plus (Dako, Denmark), following a standard protocol per the manufacturer’s recommendations. This study used a polyclonal rabbit *PTEN* antibody (Anti-*PTEN* antibody, Y18 ab32199, abcam, UK) with a 1:100 dilution. Signal detection was carried out using Dako flex dual link detection kit (secondary antibody and DAB system K 8000, Dako, Denmark). *PTEN* immunostaining was evaluated by four observers independently (AA, SA, MF, and HH), and an IHC score of *PTEN* expression was developed, as per our recent report [4]. Briefly, staining intensity was evaluated using a 4-tiered scoring system as follows: 0 – negative; 1 – weak; 2 – moderate; and 3 – marked. We considered scores 0-1 as ‘*PTEN* loss’ and 2-3 as ‘intact *PTEN*.’ Endothelial cells and stroma were used as a negative internal control, while benign prostatic glands served as an internal positive control. We have previously validated this IHC score against the confirmatory quantitative Polymerase Chain Reaction Single-Stranded Conformation Polymorphism (PCR-SSCP) mutation score, yielding a sensitivity of 58.9% and a specificity of 83.9% [4].

### Statistical analysis

Statistical analysis was performed using the Statistical Package for Social Sciences (SPSS©) software (version 19; 2010, IBM, USA). Descriptive summary statistics were used to describe the demographic and clinical characteristics of cases. Frequency and percentages were used for categorical variables. Summary means (mean ± SD) for each continuous measure was recorded. Parametric analysis was chosen for all studies assuming that data are normally distributed. Kaplan-Meir survival analysis was used to estimate the mean and median survival time (time-to-resistance) for the different ADT regimens. Log Rank (Mantel-Cox) analysis was used to compare survival proportions by ADT regimen. A p-value less than 0.05 was considered significant for all studied analyses.

## Results

### Clinical features

Table 1 summarizes the major clinical and pathologic features of the study population. The mean age (±SD) of prostate cancer patients was 73.3 ± 10.5 years (range 37-94 years). The mean baseline serum PSA was 172.6 ± 447.7 ng/mL, while the mean highest serum PSA during ADT was 242.3 ± 603.2 ng/ml. The majority of prostate cancers in our cohort were stratified into either Gleason Score (GS) 7 (29.8%) or GS9 (28.8%). However, according to the WHO-ISUP Grade Group (GG) system, most cases were in GG5 (42.3%). Three different monotherapy ADT regimens were used in our patients: goserelin (15.4%), triptorelin (13.5%), or bicalutamide (5.4%). Combination/sequential ADT, defined as the use of two or more classes of ADT concomitantly or switching from one class of ADT to another, was the most frequently utilized therapeutic strategy in the study population (66.3%). Chemotherapy was administered in 11.5% of prostate cancer cases. Approximately 61% of the prostate cancer cases in this cohort lacked *PTEN* expression, as assessed by the IHC score, with a trend of increased frequency of *PTEN* loss in WHO-ISUP GG5 cases (Table 2).

**Table 1:** Clinical and pathologic features of prostate cancer cases.

**Sample Size**	104
**Mean age (range)** Median (IQR)	73.3 (37–94) 74.0 (11.0)
**Mean baseline PSA (range) (ng/ml)** Median (IQR)	172.6 (0.02–3322) 38.9 (89.1)
**Mean highest PSA during ADT (range) (ng/ml)** Median (IQR)	242.3 (0.6–3244) 17.24 (98.0)
**Gleason Score distribution (%)**	
3+3	6.7
3+4/4+3	29.8
4+4/3+5	21.2
4+5	28.8
5+5	13.5
**WHO-ISUP Grade Group distribution (%)**	
Grade Group 1	6.7
Grade Group 2	24.0
Grade Group 3	5.8
Grade Group 4	21.2
Grade Group 5	42.3
**ADT type (%)**	
Goserelin only	15.4
Triptorelin only	13.5
Bicalutamide only	4.8
Combination/Sequential therapy	66.3
Chemotherapy (%)	11.5
***PTEN* status according to IHC Score (%)**	
Intact *PTEN*	39.4
*PTEN* Loss	60.6

PSA – prostate-specific antigen; ADT – androgen-deprivation therapy; ISUP – International Society of Urologic Pathology; IHC – immunohistochemistry.

**Table 2:** Distribution of *PTEN* expression in prostate cancer cases based on the immunohistochemistry score stratified by the Gleason Score (GS)/WHO-ISUP Grade Group (GG).

	Gleason Score (GS)/WHO-ISUP Grade Group (GG) Number (%)					
	GG 1 (GS 3+3)	GG 2 (GS 3+4)	GG 3 (GS 4+3)	GG 4 (GS 4+4/3+5)	GG 5 (GS 4+5/5+5)	Total
***PTEN* Loss**	3 (42.9)	10 (40)	6 (100)	14 (63.6)	30 (68.2)	63 (60.6)
**Intact *PTEN***	4 (57.1)	15 (60)	0 (0)	8 (36.4)	14 (31.8)	41 (39.4)
**Total**	7	25	6	22	44	104

### *PTEN* expression status and time to castration resistance

Given our previous finding that *PTEN* loss occurs in approximately 60% of prostate cancer cases in Jordan [4], we aimed to investigate whether *PTEN* expression status is correlated with time to castration resistance during ADT in this cohort. Overall, there was no significant difference in the mean time-to-resistance between patients with *PTEN* loss and those with intact *PTEN* (24.8 and 24.2 months, respectively; p=0.9). Moreover, stratifying prostate cancer cases according to the ADT regimen utilized for treatment revealed that *PTEN* expression status (*PTEN* loss vs. intact *PTEN*) had no significant effect on mean time-to-resistance in patients receiving goserelin only (p=0.19), triptorelin only (p=0.13), bicalutamide only (p=069), or combination/sequential ADT (p=0.87), as shown in Table 3.

**Table 3:** Mean time-to-resistance to ADT (months) in prostate cancer cases, stratified by the type of ADT and *PTEN* expression status.

	*PTEN* Loss	Intact *PTEN*	P
**Goserelin only**	4.9	12.6	0.19
**Triptorelin only**	17.0	5.8	0.13
**Bicalutamide only**	18.4	24.0	0.69
**Combination/Sequential therapy**	31.5	30.4	0.87
**Overall**	24.8	24.2	0.9

### ADT regimen and time to castration resistance

**Figure 1:**
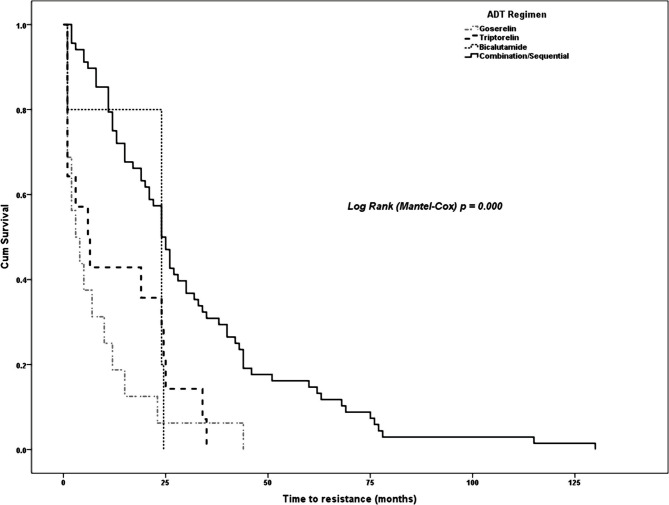
Survival analysis of time-to-resistance to ADT (months) in prostate cancer cases, stratified according to the type of ADT.

Considering that prostate cancer patients in this cohort received various ADT regimens as described earlier, we examined whether the type of ADT regimen used had an impact on patient survival, expressed as the time to castration resistance. We found that the use of combination/sequential ADT significantly delayed the onset of CRPC, with a median time-to-resistance of 24 months (95% confidence interval = 20.8 – 27.2), compared with the other monotherapy ADT regimens used in this cohort (log-rank Mantel-Cox p=0.000) as shown in Figure 1.

## Discussion

The major findings of this clinical study are (1) *PTEN* loss is not a major determinant of time to castration-resistance in prostate cancer in Jordan, and (2) combination/sequential ADT regimens achieve better outcomes of hormonal therapy compared to monotherapy regimens, by delaying the onset of CRPC. To the best of our knowledge, this is the first study conducted in the Eastern Mediterranean region to demonstrate the correlation of *PTEN* loss with response to ADT in a cohort of prostate cancer cases.

Prostate cancer is the second leading cause of cancer-related deaths in men worldwide [16], harboring massive economic and psychosocial burdens globally [17]. The most recent guidelines of the National Comprehensive Cancer Network (NCCN) recommend a more balanced approach to prostate cancer using early detection tools and active surveillance strategies based on a well-defined risk stratification scheme [18]. This scheme is based on traditional prognostic factors, such as the serum PSA, the pathologic GS and WHO-ISUP, and the clinical staging of the disease [19]. The major shortcoming of these prognostic tools is that they frequently fail to predict the disease trajectory and to accurately gauge prospective responsiveness to ADT, which remains the primary systemic therapy for metastatic prostate cancers [20]. Such limitation is thought to be related to the inherent heterogeneity of prostate cancer patients within each NCNN risk group, with a substantial body of evidence alluding to a cornerstone role of prostate cancer genetics in the ultimate outcomes of therapy [21].

Several germline and somatic mutations have been strongly implicated in the pathogenesis of prostate cancer, accounting in part for the apparent ethnic disparity in the prevalence rates of the disease worldwide [22]. Our recent study reported *PTEN* loss in approximately 60% of prostate cancer cases in Jordan [4]. Intriguingly, these rates were significantly higher than those reported in East Asian cohorts (10%-34%) and on the upper end of frequencies found in Western populations (18%-70%). The current study, therefore, takes impetus from our previous results by exploring whether *PTEN* expression status correlates with responsiveness to ADT in Jordanian prostate cancer patients.

We herein report that *PTEN* expression status does not correlate with time to castration resistance in prostate cancer. This finding contrasts a host of previous reports linking *PTEN* loss to the development of CRPC state. For instance, Ferraldeschi et al. showed in 2015 that loss of *PTEN* was associated with a shorter duration of treatment and overall survival among 144 prostate cancer patients receiving hormonal therapy [7]. Similarly, Mithal et al. (2014) reported that *PTEN* loss significantly predicted the time to the development of metastasis, CRPC, and response to ADT after radical prostatectomy [23]. Conversely, a recent study by Tabakin et al. in 2018 showed that *PTEN* expression status, among other genetic modifications, did not correlate with response to ADT [24].

On the other hand, we found a significant prolongation of median time-to-resistance in patients receiving combination/sequential ADT regimens compared to those on monotherapy regimens. This finding is consistent with a study by Akaza et al. in 2004, where they reported superior efficacy of combined ADT versus LHRH agonist monotherapy in advanced prostate cancer, with respect to time to treatment failure and time to progression [25]. In 2009, the same group reported a significant, albeit small, overall survival advantage with combined ADT regimens over a median follow-up period of 5 years in comparison to monotherapy regimens [26]. However, these findings must be discussed in the broader context of the risk/benefit ratio, considering adverse events and the cost of the combined regiments [27]. The utilization of sequential ADT in our institution follows the current guidelines of the European Association of Urology - European Society for Radiotherapy & Oncology - International Society of Geriatric Oncology (EAU-ESTRO-SIOG) on prostate cancer. These guidelines recommend a bi-annual evaluation of ADT effectiveness in maintaining a castration level, switching to another type of ADT, or adding an antiandrogen if the utilized ADT proved inadequate [28].

The choice of time to castration resistance as an endpoint in our study is based on a host of recent studies that have indicated its important prognostic value as a novel predictor of overall prostate cancer survival. For instance, Hakozaki et al. showed in 2022 that time to CRPC is a significant predictor of cancer-specific survival in patients with nonmetastatic castration-resistant prostate cancer [29]. The same was earlier reported by Bournakis et al. in 2011 [30]. Moreover, Miyake et al. reported in 2019 that time to castration resistance is independently correlated with the overall survival of patients with metastatic castration-sensitive prostate cancer [31].

This study has several limitations. First, the sample size is not sufficient to yield a more robust analysis with more precise correlation statistics between *PTEN* expression status and response to hormonal therapy. Second, our primary and secondary outcomes did not include patient survival, owing to the absence of accurate institutional patient records and population-based registries. However, we are genuinely working on setting up a national registry for prostate cancer in Jordan, which should address these weaknesses. Third, our patient population with prostate cancers is older than that in similar studies and likely demonstrated more advanced disease, which could theoretically bias the interpretation of our data in relation to existing literature, as the prevalence of *PTEN* loss is higher than that reported in similar cohorts from the region and on the upper end of rates reported from Western countries. Despite these limitations, the novel prognostic value of the presented results warrants further investigation in future clinical trials.

## Conclusions

Our study provides evidence that *PTEN* loss is not a major determinant of time to castration resistance following ADT in a cohort of prostate cancer patients in Jordan. The use of combination/sequential ADT seems to procure a significant therapeutic advantage over monotherapy regimens, delaying the onset of CRPC. Future clinical studies exploring the long-term effectiveness and safety profiles of these two regimens are warranted.

## Data Availability

The clinical data used to support the findings of this study are included in the article.

## Personal thanks

The authors thank Dr. Mahmoud Alfaqih at Jordan University of Science and Technology for his editorial help in reviewing the manuscript.
